# Draft Genome Sequence of the Methicillin-Resistant *Staphylococcus aureus* Isolate MRSA-M2

**DOI:** 10.1128/genomeA.00037-12

**Published:** 2013-01-31

**Authors:** Janette M. Harro, Sean Daugherty, Vincent M. Bruno, Mary Ann Jabra-Rizk, David A. Rasko, Mark E. Shirtliff

**Affiliations:** a Department of Microbial Pathogenesis, School of Dentistry, University of Maryland, Baltimore, Maryland, USA; b Institute of Genome Sciences, University of Maryland, School of Medicine, Baltimore, Maryland, USA; c Department of Microbiology and Immunology, School of Medicine, University of Maryland, Baltimore, Maryland, USA; d Department of Oncology, Diagnostic Sciences, School of Dentistry, University of Maryland, Baltimore, Maryland, USA

## Abstract

We report the draft genome sequence of a methicillin-resistant strain of *Staphylococcus aureus*, designated MRSA-M2. This clinical isolate was obtained from an osteomyelitis patient undergoing treatment at the University of Texas Medical Branch (Galveston, TX). This strain is an ST30, *spa* type T019, *agr* III strain and has been utilized as a model *S. aureus* strain in a number of proteomic, transcriptomic, and animal model studies.

## GENOME ANNOUNCEMENT

*Staphylococcus aureus* is among the leading pathogens causing bloodstream infections able to form biofilms on host tissue and indwelling medical devices and to persist and cause disease (1). Infections caused by *S. aureus* are becoming more difficult to treat due to an increasing resistance to antibiotics. In addition to genetic mechanisms of antibiotic resistance, *S. aureus* isolates often have the ability to form biofilms, making them even more resistant to treatment (2). *S. aureus* M2 has been used as a model *S. aureus* strain in a number of proteomic, transcriptomic, and animal model studies (3–9) and has shown interkingdom interactions between *S. aureus* and *Candida albicans* (10, 11) and the potential importance of quorum sensing in pathogenesis of both organisms in animal model hosts (10, 11).

The *S. aureus* M2 strain was obtained from an osteomyelitis patient undergoing treatment at the University of Texas Medical Branch (Galveston, TX) (3, 9). This strain is an ST30, *spa* type T019, *agr* III strain. Following isolation, the strain was stored at −70°C in defibrinated sheep blood and grown on tryptic soy broth (TSB) and tryptic soy agar prior to genomic DNA extraction. Genomic DNA was isolated from an overnight culture using the Qiagen genomic-tip Sigma GenElute kit (Qiagen) with the addition of lysostaphin (100 µg/ml) and incubation at 37°C for 1 h in the initial step (Sigma-Aldrich). The genomic DNA was sequenced at the University of Maryland School of Medicine, Institute for Genome Sciences, Genome Resource Center (http://www.igs.umaryland.edu/). The genome sequence of *S. aureus* M2 was generated using paired-end libraries with 300-bp inserts on the Illumina HiSeq2000. The draft genome data were assembled using the Velvet assembly program (12). The resulting genome assembly contained 133 contigs that were longer than 500 bp. This results in a predicted genome size of 2,902,463 bp with an average G+C% of 32.12 (range 25.69 to 51.17%). Both of these parameters are similar to those reported for previous *S. aureus* genome projects (http://gscid.igs.umaryland.edu/wp.php?wp=03genome_analysis_of_the_staphylococcus_aureus_complicated_infection_group). The contig data were annotated using the Annotation pipeline at the Institute for Genome Sciences, Informatics Resource Center (http://www.igs.umaryland.edu/). The predicted genes from the draft genomes were also similar to previously sequenced *S. aureus* genomes, with 2,725 genes. This predicted gene count is similar to those of other *S. aureus* genomes in the public domain.

The genomic features associated with the previously observed phenotypes of the M2 isolate, such as sequence type ST30, *spa* type T019, and an *agr* III phenotype, were each identified and verified in the draft genome sequence. The *icaADBC* operon was also confirmed in the sequence. The *icaADBC* locus mediates poly-*N*-acetyl-β-1,6-glucoamine assembly into the polysaccharide intercellular adhesin (13) that regulates biofilm formation in multiple *S. aureus* strains (14). Isolates of the *agr* III phenotype, which lack a functional *agr* system, are identified as medium biofilm producers due to *icaAR* and *rsbU* expression during late- and postexponential growth phases (15).

Further molecular-based studies are under way with the *S. aureus* M2 isolate that will advance our understanding of *S. aureus* pathogenesis.

### Nucleotide sequence accession number.

The genome data have been deposited in GenBank with accession number AMTC00000000.
